# Acute Genetic Manipulation of Neuronal Activity for the Functional Dissection of Neural Circuits — A Dream Come True for the Pioneers of Behavioral Genetics

**DOI:** 10.3109/01677063.2012.663429

**Published:** 2012-03-15

**Authors:** Moto Yoshihara, Kei Ito

**Affiliations:** 1 Department of Neurobiology, University of Massachusetts Medical School, Worcester, Massachusetts, USA; 2 Institute of Molecular and Cellular Biosciences, University of Tokyo, Tokyo, Japan

**Keywords:** behavioral genetics, *Drosophila*, optogenetics, thermogenetics

## Abstract

This review summarizes technical development of the functional manipulation of specific neural circuits through genetic techniques in *Drosophila*. Long after pioneers ’ efforts for the genetic dissection of behavior using this organism as a model, analyses with acute activation of specific neural circuits have finally become feasible using transgenic *Drosophila* that expresses light-, heat-, or cold-activatable cation channels by xxx/upstream activation sequence (Gal4/UAS)-based induction system. This methodology opened a new avenue to dissect functions of neural circuits to make dreams of the pioneers into reality.

## INTRODUCTION

From late 1960s, Hotta and Benzer started genetic dissection of behavior controlling mechanisms with classic genetic methodology, that is, mutant analyses through behavior observation. Such mutant analyses had made great success in genetics of bacteria and phages. Likewise, mutant analyses using *Drosophila* made a substantial success to understand functions of the nervous system. However, there was a certain caveat in this approach. Complex behavior of a given multicellular animal is regulated by a neuronal network consisting of thousands of neurons connected with each other. We cannot expect that the function of a single gene should regulate the entire pattern of certain behavior. Thus, disruption of a single gene in all the neurons would not provide enough insights for understanding the mechanisms understanding the control of behavior through complex neuronal network.

Therefore, success in *Drosophila*'s behavioral genetics was restricted to only a limited repertoire of genes, whose specific defects result in a global behavioral disorder. One great example of such success was the analysis of the *period (per)* gene, which was identified as causing defects in circadian rhythm by Konopka in Benzer's lab (Konopka & Benzer, 1971). Another representative example, *Shaker (Sh),* was identified for its phenotype of ether-induced tremor (Kaplan & Trout, 1969) and turned out to be a structural gene of potassium channel (Kamb et al., 1987; Papazian et al., 1987; Tempel et al., 1987). In both *per* and *Sh,* phenotypes of the mutant genes were clearly recognizable as behavior defects of a whole animal. Although mutants isolated from behavior screening gave certain useful information, we had some kind of frustrated feeling that behavior genetics had not given fundamental mechanistic insights into the regulation mechanisms of complex behavior. Attempts for genetic dissection of behavior had not really dissected the role of the complex neural networks in the brain. Rather, they tend to result in the dissection of other issues such as intracellular signal transduction or fate determination during development.

Recently, a technical breakthrough that enables acute activation, i.e., “remote control” of specific neurons, was developed using *Drosophila* genetics (Lima & Miesen-bock, 2005), which subsequently applied in other model organisms such as mice (Zhang et al., 2006). The new approach has opened up a way to dissect functions of neural networks. In this review we discuss early attempts of genetic dissection of behavior in *Drosophila* and subsequent advancement of genetic manipulation during three decades of *Drosophila* neurogenetics. These studies were prerequisites for the recent genetic activation approach. We then discuss what in behavior we can learn using these modern techniques, taking our own recent attempt as an example. Because there are many comprehensive reviews of remote controlling technique (Fenno et al., 2011; Miesenbock, 2009), we aim mainly at describing historical meaning of the techniques, focusing on pioneers’ efforts to discuss what they tried to reveal and what kinds of technical developments during past three decades led to the breakthrough.

## EARLY ATTEMPTS FOR THE GENETIC DISSECTION OF BEHAVIOR

### Seymour Benzer's Approach — Hotta's Dream

Gregor Johann Mendel, former student of a physicist Christian Andreas Doppler at the University of Vienna, adopted quantitative methodology of physics into biology to analyze the mechanisms of inheritance by counting populations of peas (Mendel, 1866), leading to the establishment of “genetics.” In a similar quantitative strategy, Seymour Benzer at the California Institute of Technology (Caltech), also a former physicist who then performed a historical study in bacteriophage genetics to establish the concept of “cistron,” began genetic analyses of *Drosophila* behavior by observing a population of organisms rather than individual animals to quantify phenotypes. The first example of Benzer's challenge was the quantification of phototaxis behavior with the “counter-current apparatus” (Benzer, 1967). As the Benzer's first postdoctoral fellow for *Drosophila* research, Yoshiki Hotta isolated many mutants in visual behavior using this technique. To characterize those visual mutants, Hotta employed elec-troretinogram (ERG), which is the extracellular potential recording on the surface of *Drosophila* compound eyes (Hotta & Benzer, 1969). Their approach was proven to be quite powerful to dissect various phenomena in neuro-science. For example, Hotta and Benzer's screening has led to the finding of many important mutants in signal transduction of *Drosophila* visual system such as *norpA,* which encodes phospholipase C protein (Bloomquist et al., 1988; Masai & Hotta, 1991).

### Ikeda's Dream—“Command Neuron” in Neuroethology

Interestingly, another Japanese scientist, Kazuo Ikeda, also started genetic dissection of *Drosophila* behavior at the same time. He approached in the opposite direction to that of Hotta and Benzer. Whereas the latter moved from genetics to behavior, Ikeda originally worked in the field of neurophysiology and neuroethology, from which he sought involvement of genetic factors in behavior. Before using *Drosophila* as a model, Ikeda had made a historically important study in neuroethology: the finding of “command neurons.” In Wiersma's lab at Caltech, Ikeda recorded periodic bursting pattern of ventral ganglion of crayfish that correspond to the movement of its swimmeret (Ikeda & Wiersma, 1964). Then, Ikeda physically dissected a crayfish neuropil connecting ganglions, and stimulated neurons one by one to find neurons that can change the bursting pattern. After tiresome efforts, Ikeda found specific neurons that can turn *on* or *off* the bursting pattern, which he named the “command neurons” (Wiersma & Ikeda, 1964). It was the birth of the concept of command neurons, which was revived by a recent technical breakthrough we explain later.

After this study Ikeda embarked on the study of command neurons in *Drosophila* to seek genetic regulation of commanded behavior. For this purpose, He chose the fly's flight system as a model (Ikeda et al., 1980; Koenig & Ikeda, 1980a, 1980b). Although physiological studies of the *Drosophila* flight system, together with those by Wyman's group (Harcombe & Wyman, 1977; Tanouye & Wyman, 1980), gave certain insights on the understanding neuronal networks, unfortunately we cannot say that genetic methods have provided sufficient data to understand the functional regulation of the flight system. Because genetic methodology had not yet developed sufficiently at that time, Ikeda and his colleagues of the period could not make full use of genetic technique for dissecting neural networks. Rather, because fruit flies are so small, fine neurophysiology at the same level as those performed at crayfish proved to be difficult.

Ikeda's interest shifted towards synaptic function using neuromuscular junctions of flight muscles as model synapses, leading to the series of works on *shibire* mutant. This mutant was isolated from a series of forward genetic screening of temperature-sensitive paralysis performed by David Suzuki and colleagues (Grigliatti et al., 1973). The temperature-sensitive *shibire^ts^* mutant enabled acute temporal controlling of its phenotype, providing clear data for their physiological experiments. This study led to the first genetic dissection of synaptic function (Ikeda et al., 1976) and a critical finding that *shibire* mutant has defects in the recycling step of synaptic vesicles (Kosaka & Ikeda, 1983). Together with the cell biological analysis of Dynamin, the protein coded by the *shibire* gene (Takei et al., 1995), these analyses greatly contributed to the understanding of vesicle trafficking.

Thus, Ikeda's *shibire* study started from the dissection of specific behavior but resulted in the understanding of general cell biology. Interestingly, though, the *shibire^ts^* mutant became a critical tool for acute temporal regulation of specific neurons more than 30 years later, bringing a methodological breakthrough for the functional dissection of neural circuits that Ikeda originally intended to address (see below).

### Mosaic Analyses by Ikeda and Hotta

Hotta's forward genetics approach and Ikeda's physiological approach paved the way to understanding the relationship between genes and physiological phenomena. For example, Ikeda and Kaplan made the first extracellular and intracellular recording from the *Drosophila* central nervous system to show abnormal bursting patterns of action potential in the thoracic ganglion of *Hyperkinetic (Hk)* mutant flies (Ikeda & Kaplan, 1970a). This study ties with the first genetic dissection of *Drosophila* elec-trophysiology using ERG recording by Hotta and Benzer (Hotta & Benzer, 1969).

To ask which cells are actually responsible for the defect, Ikeda and Kaplan (Ikeda & Kaplan, 1970b) employed a genetic tool called mosaic analyses. The technique takes advantage of the ring X chromosome, which is lost during the first nuclear division to make a gynandromorph: an animal one half of which is made from male cells and the other half from female cells. A heterozygous egg with a mutant gene on the X-chromosome and otherwise wild-type ring X chromosome, after loss of the ring X, becomes an adult gynandromorph fly with mutant hem-izygous male cells and heterozygous female cells, the latter showing wild-type phenotype. By correlating bursting phenotype and external markers in gynandromorphs, they found that abnormal bursting activity by Hk in one side of the thoracic ganglion corresponds to the external markers linked to Hk mutation, on the closest leg, suggesting that the bursting phenotype is cell autonomous to the *Hk* mutation. Hotta and Benzer (Hotta & Benzer, 1970) also performed mosaic analyses on an ERG mutant, *tan,* and suggested that the abnormal ERG is cell autonomous to the mutation.

In these mosaic studies, the observed behavior can be explained by the function of single cells because of their cell autonomous nature, and it is the reason why the mosaic analyses worked nicely. It is more difficult to utilize single gene mutation to dissect complex behavior that depends on the synergistic activity of complex neural networks, because it would not be common that a single gene is specifically responsible for the expression of a complex behavior in which various types of neurons are involved. More neurophysiological genetic tools are required to address these issues.

### Hotta's Courtship Analyses—A Milestone of Classical Genetic Dissection

Hotta proceeded one-step forward by plotting the responsive locus of various behavior mutants onto the blastoderm map they newly established (Hotta & Benzer, 1972). In 1976, Hotta and Benzer made the first success in dissecting complex behavior (Hotta & Benzer, 1976). Though they employed the same mosaic analyses technique, their approach this time was different from the previous ones; the study did not deal with any mutant gene or mutant phenotype, instead it dealt with the behavior that is different between male and female, namely the male-specific courtship behavior. Instead of using flies with any mutant gene on the X-chromosome, they just made gynandromorphs and observed their courtship. If the cells responsible for male courtship behavior are male cells in a gynandromorphous fly, it should behave as a male, and vice versa. These analyses allowed them to determine which region in a fly's body is responsible for courtship behavior. Through the mosaic analyses, they found that the region responsible for the male's wing vibration behavior to attract a female is located in the brain, whereas the region responsible for the next step of courtship, attempting copulation, resides in the thoracic ganglion.

In this special case, male/female difference in certain parts of the body caused clear behavioral differences, even though underlying structural/functional changes in the neural network should be very complex. Their courtship mosaic analysis was extremely cool and unique in this regard, and has a historical importance as the first clear genetic dissection of a network-dependent complex behavior.

However, because this mosaic analysis maps the focus of responsible cells by measuring the correlation with the morphological phenotype of the external markers, the spatial resolution was rather low. Though it was able to map the responsible sites of wing vibration and attempting copulation behaviors onto the brain and the thoracic ganglion, it was not possible to locate which neurons in these ganglia are actually involved in courtship control. We had to wait until recent technical advances for enabling more detailed analyses at the network level. In the following sections, we explain technical advancements that fruited into the present genetic manipulations of behavior at single-cell level.

## DEVELOPMENT OF THE GENETIC MANIPULATION TOOLS FOR NEURAL CIRCUIT ANALYSIS

### Development of Gene Expression System in *Drosophila* Molecular Genetics

One of the most influential breakthroughs in fly genetics was the transformation technique of ectopic genes using the P element transposon, developed by Rubin and Spradling (Rubin & Spradling, 1982; Spradling & Rubin, 1982). It enabled researchers to introduce any gene of interest into fly genome to drive its expression. As an additional benefit, the technique turned out also to be highly useful for mutagenesis, because the P element transposon is randomly inserted into the fly genome to cause an in-sertional mutation. Intentional mobilization of P element transposon by supplying the transposase source was attempted for the purpose of mutagenesis, followed by the cloning of genes adjacent to the P-element insertion (Cooley et al., 1988; Yoshihara et al., 1988). A transfor-mant strain often called “delta2 - 3,” in which a P element transposase gene without the intron between open reading frames ORF2 and ORF3 (Laski et al., 1986) was stabilized in the genome (Robertson et al., 1988), turned out to be the most stable transposase source, which is used as a common tool for P element-mediated transformation and mutagenesis until today.

The next important step in the history of *Droso-phila* genetics appeared soon afterwards: the “enhancer trap” technique (O'Kane & Gehring, 1987). Because the original P element-mediated mutagenesis mobilized a transposon that carries only a marker gene to isolate the transgenic flies (e.g., *rosy* or *white* driven by a constitutive promoter), the recombinant line remained useless unless the transposon was inserted right into or near certain gene to induce detectable phenotype. O’ Kane put one more gene construct called “reporter” in the transposon. The reporter is a gene whose expression can easily be detectable—e.g., the β-galactosidase gene *lacZ*—, under the control of a weak promoter. The reporter gene will be expressed under the influence of a nearby enhancer. Thus, the activity of the enhancer near the insertion locus can be “trapped” by visualizing the reporter protein. If the enhancer regulates the expression of an endogenous gene A, the expression pattern of the reporter gene is supposed to mimic the expression of gene A. The enhancer-trap strains provided an efficient way to isolate genes with specific expression pattern regardless of whether the transposon caused inser-tional mutation.

Another big advancement came several years later by Brand and Perrimon (Brand & Perrimon, 1993), who introduced a yeast transcription factor, *Gal4,* and its target, the upstream activation sequence (UAS), into fly genome. Unlike reporter genes such as *lacZ, Gal4* does not visualize the cells by itself but drives expression of another gene that is put downstream of UAS. By crossing a single strain carrying a Gal4 insertion with the strains carrying various genes under UAS, these genes can be expressed in the same set of cells reproducibly. Likewise, by crossing a strain carrying a UAS construct with various *GAL4-*carrying strains, the same gene can be expressed specifically in numerous ways. The separation of the tools to specify the gene expression pattern (by *Gal4* strains, called drivers) and those for selecting the genes to be expressed has dramatically enhanced the way to visualize and manipulate specific cells in the nervous system, fulfilling a requirement for the modern approach towards circuit dissection.

### Effectors for Neuronal Function: Spatially Regulated Neural Manipulation

The UAS-conjugated genes are called either a reporter or effecter, depending on whether it is used just to visualize the cells or to alter their fate or function. Taking the advantage of the Gal4/UAS system, many effecter genes were developed. For example, Sweeney et al. (Sweeney et al., 1995) made *UAS-tetanus toxin light chain (TNT),* which cleaves a synaptic vesicle protein Synaptobrevin that is essential for nerve-evoked synaptic transmission (Yoshihara et al., 1999). There were also attempts to suppress or enhance activity of neurons. To suppress activity of neurons by enhancing potassium conductance, White et al. (White et al., 2001) made *UAS-EKO* (electrical knock out) by genetic engineering of the *Shaker* potassium channel (Kamb et al., 1987; Papazian et al., 1987; Tempel et al., 1987) to deprive its inactivation property. Baines (Baines et al., 2001) made UAS-Kir, which encodes an inward rectifier potassium channel that regulates the resting potential of neurons. Overexpression of *Kir* channel is also supposed to increase potassium conductance, leading to the inactivation of neurons. In the opposite direction, *UAS-NaChBac,* which is a bacterial sodium channel, was introduced into fly genome to enhance neural activity by increasing sodium conductance (Luan et al., 2006a). Using these effectors, researchers can now block synaptic transmission (by UAS-TNT) or suppress or enhance neural activity (by UAS-driven *EKO* and *Kir* or *NaChBac,* respectively) of specific sets of neurons.

### UAS-*shibire:* Breakthrough for Temporary Regulated Neural Manipulation

However, the effect of these manipulations are chronic; the nervous system may resort to physiological or developmental compensatory effects to minimized the defects caused by the malfunctioning neurons (Turrigiano & Nelson, 2000). Thus behavioral phenotypes by expression of these effecters are somewhat tricky to be interpreted.

After a series of seminal study on the *shibire* mutant in synaptic transmission by Kazuo Ikeda, Kim and Wu (Kim & Wu, 1990) found antimorphic effect of the mutation, *shibire^ts1^,* that is, even heterozygous *shibire^ts1^*/+ flies show weak paralytic phenotype. It suggested that the expression of the mutant gene works dominantly to suppress synaptic transmission. To take advantage of this dominant effect, Toshi Kitamoto, who was working in the next lab to Kazuo Ikeda, simply combined UAS with *shi-bire^ts1^,* which had been cloned by Meyerowitz's lab (van der Bliek & Meyerowitz, 1991). Overexpressed mutant *shibire* protein caused no effect when the flies are kept in low temperature, but quickly suppressed synaptic transmission when the ambient temperature was raised to ca. 30°C (Kitamoto, 2001). This system provided researchers with a highly efficient tool to manipulate neural function both cell-specifically and temporally. The system became immediately popular as a genetic tool for memory studies to prove its efficiency (Waddell et al., 2000; Dubnau et al., 2001; McGuire et al., 2001). The ease of temporal regulation just by temperature shift allowed researchers to ask when synaptic transmission from specific groups of neurons is required for each step of memory formation and retrieval.

(Note: we should be careful when interpreting the results of *shibire* experiments. Because *shibire* is a mutant form of Dynamin GTPase, a crucial protein for vesicle formation, its malfunction not only affects synaptic vesicles but also intracellular vesicle trafficking in other parts of the cells. For example, effect of *shibire* is also observed in the postsynaptic compartment, which may well play important roles in memory formation [Yoshihara et al., 2005], and expression of *shibire* in the glial cells blocks their phagocytic action to affect axon pruning [Awasaki & Ito, 2004]).

### Temperature-Sensitive Gal80: An Alternative Approach for Temporary Regulated Neural Manipulation

As discussed before, there are many effecter genes that can block or alter neural function, but unfortunately temperature-sensitive mutant variants have not been identified for them. To compensate this problem, another method for spaciotemporal neural manipulation was developed using a temperature-sensitive allele of *Gal80,* which inhibits Gal4 function by its binding to Gal4 in yeast (Ma & Ptashne, 1987). The ability of Gal4 as cell-specific expression driver can be blocked by expressing *Gal80^t^s* which is usually driven generally by constitutive promoter such as *Tubulin* promoter. When the temperature is raised to ca. 30°C, the temperature-sensitive GAL80^ts^ protein can no longer suppress GAL4, allowing the expression of UAS-conjugated effecter genes (McGuire *et al.,* 2003).

When the flies are kept at high temperature for a relatively long period (e.g., overnight), the effecter protein accumulated in the cells can continue affecting the neural function for several hours after the flies are moved back to the ordinary temperature. This is convenient in certain experiments in which normal behavior is affected simply by raising the temperature (e.g., auditory courtship response; Kamikouchi et al., 2009). In those experiments, chronic temporal regulation using *Gal80^ts^* has advantage over acute manipulation using UAS-*shibire.*

### Acute Activation of Ion Channels: The Biggest Breakthrough to “Remote Control” Neurons

Although effecters such as *shibire^ts^, TNT, EKO, Kir,* and *NaChBac* block or modify neural function, it is not possible to activate specific neurons acutely like stimulating electrode can do. A new series of technical breakthrough to address this issue came in three flavors: activation of cation channels by caged compound, light, and temperature shift.

The first approach was developed by the laboratory of Gero Miesenbock (Lima & Miesenbock, 2005). They made a transgenic fly carrying the UAS construct with the mammalian adenosine triphosphate (ATP) receptor channel gene, whose homologue does not exist in fly genome. To activate the channel, they injected caged ATP into fly's body. Flashing light uncaged the caged molecule to release ATP, which then binds to the ATP receptor to open the channel, causing influx of cations into neurons to depolarize them. To test the system, they expressed the ATP receptor channel in the giant fiber neurons, which are known to trigger the jump muscle of the legs to induce escape response. As expected, light illumination triggered flies to suddenly jump and beat wings. As they called the methodology “remote control,” the new technique can activate neurons in free-running animals, a great advantage over the conventional stimulation methods with electric wires used for mammals. The dramatic success of Lima and Miesenbock revived Ikeda's concept, the “command neuron,” which triggers a stereotypic pattern of behavior such as the escape response commanded by the giant fiber neurons. However, administration of caged ATP into tiny fly bodies is not very practical, making it difficult to apply this technique to a large number of animals.

Another approach made use of a bacteria-derived Channelrhodopsin 2 (ChR2), a sodium channel that opens when illuminated by blue light (Nagel et al., 2003). Unlike ATP receptor channel, injection of additional chemical is not required. A flush of blue light is enough to activate the ChR2-expressing neurons. The system was successfully used in fly larvae to activate different types of monoamine neurons to induce reward and avoidance learning (Schroll et al., 2006), showing that these neurons convey essential signal for memory formation. Because light stimulation is easily applicable to vertebrates, the technique is now commonly used by mammalian researchers (Zhang et al., 2006). However, remote control with ChR2 is rather difficult for adult flies because of the two reasons. First, unlike larvae, the adult fly is covered by brownish cuticle, which prevents efficient transmission of blue light into the brain.

And second, even though ChR2 does not require injection of caged chemicals, it does require retinal for its activation (Nagel et al., 2003). Feeding flies and worms with retinal is therefore necessary (Nagel et al., 2005; Schroll et al., 2006), whereas supplying retinal is not necessary for mammalian neurons (Boyden et al., 2005). However, whereas larvae feed ravenously, adults live on tiny amount of daily food, making it difficult to administrate sufficient amount of retinal.

The third approach utilizes heat or cold stimulus to activate neurons. *Drosophila* TrpA1 (Hamada et al., 2008) and mammalian TRPM8 (Bautista et al., 2007) are members of the ubiquitous transient receptor potential channels that respond to specific ranges of temperature to be used for fly thermoenetics. In *Drosophila,* TrpA1 is activated at high temperature (> 28°C; Hamada et al., 2008) and TRPM8 at low temperature (< 15°C; Peabody et al., 2009). Remote control can be realized by expressing these channels in specific neurons of the brain and shifting the temperature of a small chamber housing the flies. Because of the small thermal capacity of the flies due to their featherweight body mass (ca. 1 mg), heat or cold stimulation can activate neurons quickly even when they are embedded deeply in the fly brain. Thus, the temperature-induced Trp channels have become the most popular method for remote controlling neurons in the adult flies (Aso et al., 2010; Krashes et al., 2009). The technique, however, is not easily applicable to mammals because of their homeo-thermic nature and large thermal capacity.

### Methodology to Restrict Expression Beyond Enhancer Trapping

Whereas the temporal control technique of effecters has dramatically been improved during the last decade, the resolution of the spatial control remains largely unchanged since early 1990s, when the Gal4 enhancer-trap system was developed. To drive expression in a smaller numbers of neurons, a group at the Janelia Farm research campus established a large number of transformant strains in which the Gal4 fused with a small genomic fragment from an upstream or downstream region, or an intron of various genes are inserted at a fixed position of the genome (Pfeiffer et al., 2008). The expression pattern of Gal4 is expected to mimic subset of that of the endogenous gene.

However, in situ RNA hybridization and antibody labeling of many genes show a general tendency that the expression patterns of endogenous genes are by themselves not highly restricted. Rather, a gene is often expressed in a variety of cells scattered in the brain (the law of low specificity), and not all the cells in a defined brain region express the same gene (the law of low ubiquity; Ito et al., 2003). To improve the specificity of driver expression in such circumstances, so called “intersection” approach is being developed. This method makes use of two different enhancer trap patterns to induce expression only in the overlapping regions.

One method toward this aim is called the split Gal4 system (Luan et al., 2006b), in which two major domains of the Gal4 protein—the Gal4 DNA-binding domain (GAD) and Gal4 transcription-activation domain (GTA)—are put into deferent P-element constructs to make independent enhancer-trap strains. Gal4-mediated expression of UAS-linked genes should occur only in the cells where GAD and GTA are co-expressed. Thus, by crossing the GAD-enhancer trap and GTA-enhancer trap strains, specific expression can be induced only in the intersection of the expression patterns of the two lines.

Another intersection approach uses aforementioned Gal80 to inactivate Gal4 in certain cells. Enhancer-trap strains were generated with Gal80 under control of a weak promoter (Bohm et al., 2010). If one of these lines is crossed to a Gal4 line, Gal4 cannot drive expression in the cells that express Gal80. Thus, the collection of the cells that express Gal80 will be subtracted from that of the Gal4-expressing cells to attain a more specific pattern. Both split-Gal4 and Gal80-subtraction methods requires generation of a large number of new enhancer-trap strains to make the approach sufficiently versatile.

Random loss of Gal80 suppression using MARCM (Mosaic analysis with a repressible cell marker) (Lee & Luo, 1999) or flip-out Gal80 (Struhl & Basler, 1993) technique can also limit the expression pattern of Gal4. Both features yeast-derived flippase gene to induce somatic recombination or flipping-out to remove Gal80 from the DNA. If flippase is expressed only mildly, the loss of Gal80 occurs only in a few cells, in which GAL4-mediated effecter expression should occur. Unlike other methods, expression of the effecter genes is not reproducible, because the loss of Gal80 occurs only randomly. In spite of this, the approach has a potential to dissect a given neural network at the level of single neurons.

## EXAMPLES OF GENETIC DISSECTION OF NEURAL CIRCUITS BY ACUTE ACTIVATION

### Foci and Clusters: Courtship Analyses From Hotta to Yamamoto and Dickson

Following the pioneering study on *Drosophila* courtship by Hotta and Benzer (1976), Daisuke Yamamoto and his colleagues performed a large-scale screening of P-element insertion lines to isolate mutants in sex-specific courtship behavior. One of the isolated lines caused interesting homosexual behavior, which later turned out to be an allele of the *fruitless (fru)* gene, a critical regulator of male/female courtship (Ito et al., 1996). Because the original strain featured the first-generation P-element construct without any reporter or Gal4, advanced analysis of expression pattern was difficult. In what can be called bonanza, when a large scale generation of the Gal4 enhancer-trap strains by the Japanese NP (Nippon, Japan) consortium was performed with the crucial assistance of the Yamamoto lab (Yoshihara and Ito, 2000), the 21st strain out of over 4500 lines generated hit the locus of *fru,* paving the way for detailed visualization and manipulation of *fru* -expressing neurons.

Further analysis of fru-expressing neurons led Ya-mamoto's group to identify the first sexually dimorphic neurons in the *Drosophila* brain (Kimura et al., 2005). The fru-expressing neurons form several clusters. To ask which cluster is involved in triggering male courtship behavior, they employed the remote control approach to activate one of the fru-expressing neurons randomly by restricting the expression of TrpA1 effecter to singles cells by MARCM technique (Kohatsu et al., 2011) and isolated the most likely neuron clusters, P1 and P2b, which contains the sexually dimorphic neurons identified earlier (Kimura et al., 2008).

Approaching from a different starting point, the group led by Barry Dickson employed the intersection method to restrict fru-expressing neurons and identified P1 and another cluster similar to P2b as the command centers for courtship song (von Philipsborn et al., 2011). They also identified neurons in the thoracic ganglion as parts of the central pattern generators for wing vibration, whose existence had been suggested by Miesenbock's group (Clyne & Miesenbock, 2008).

Hotta and Benzer (Hotta & Benzer, 1976) identified various regions of the brain and the thoracic ganglion that are responsible for the phenotypes of each step of *Drosophila* courtship behavior and called them foci. More than 30 years later, these recent studies appear to be on the extrapolation of Hotta's mosaic analyses of courtship behavior, because the clusters of neurons identified in these studies are likely to correspond to the foci identified by their predecessor.

### Fulfilling Ikeda's Dream: The First Systematic Screening of Command Neurons

Though Ikeda established the concept of command neurons, technical limitation made it difficult to isolate such neurons in the insect nervous system. With the array of advanced molecular genetic techniques at hand, we are now in a position to make his dream reality.

The analysis of fru-regulated courtship circuitry is a sort of reverse-genetics approach, in which both the genes to be analyzed and the phenotype to be observed are defined beforehand. Considering the highly limited level of our current knowledge and understanding about brain and behavior, forward genetics approach may also provide important insights.

To this aim, we used the collection of Gal4 enhancer-trap lines established by the NP consortium (Yoshihara & Ito, 2000) to systematically seek command neuron circuits. We crossed Gal4 driver lines to cold- or heat-activatable TRPM8 (Peabody et al., 2009) or TrpA1 (Hamada et al., 2008)) channels, and video-recorded the behavior of flies after acute temperature shift to activate the channels in a small temperature-controlled chamber. Though the screening is still continuing, various elements of behavior, such as jumping, grooming, wing beating, egg laying, etc., have already been observed.

Stimulation of one strain evoked the entire sequence of feeding behavior, complete with proboscis extension, expansion of labellum, movement of pharyngeal pumps, and retraction of the proboscis. Narrowing down the responsible neuron by the “flip-out Gal80” led us to identify a single pair of interneuron neurons, named “feeding neurons,” whose activation alone can trigger the entire feeding sequence. Because this command neuron is likely to integrate information of food taste with other inputs to trigger feeding conditionally, analysis of these neurons would serve as a model system expand our knowledge of the synaptic plasticity revealed at the neuromuscular junction (Yoshihara at al., 2005) to understand the role of central synapses in the plastic neural task such as Pavlov's classical conditioning at central synapses.

Similar large-scale screening of command neurons is currently going on in various research groups. Forty-eight years after his study of crayfish, Ikeda's dream not only came reality but also became the main stream of modern neuroscience in the 2010s.

## CONCLUSION

Yoshiki Hotta preached us at his retirement at the University of Tokyo, based upon his deep insight: “We cannot change the past, but we can build our future.” Hotta and Benzer's approach made a conceptual breakthrough to understand cellular functions of neurons as the basis of behavior, which has guided scientists from various backgrounds during the last several decades. However, Hotta's dream and Ikeda's dream—to dissect behavior from the viewpoint of individual neural circuits—were somewhat too advanced at that period. To enable efficient genetic dissection of behavior, they—and we—had to wait until today's technical development. We are now thrilled to witness the dramatic future of behavioral genetics. Their dreams have just started to bloom.

## References

[b1] Aso Y, Siwanowicz I, Bracker L, Ito K, Kitamoto T, Tanimoto H (2010). Specific dopaminergic neurons for the formation of labile aversive memory. Curr Biol.

[b2] Awasaki T, Ito K (2004). Engulfing action of glial cells is required for programmed axon pruning during *Drosophila* metamorphosis. Curr Biol.

[b3] Baines RA, Uhler JP, Thompson A, Sweeney ST, Bate M (2001). Altered electrical properties in *Drosophila* neurons developing without synaptic transmission. J Neurosci.

[b4] Bautista DM, Siemens J, Glazer JM, Tsuruda PR, Basbaum AI, Stucky CL, Jordt SE, Julius D (2007). The menthol receptor TRPM8 is the principal detector of environmental cold. Nature.

[b5] Benzer S (1967). Behavioral mutants of *Drosophila* isolated by countercurrent distribution. Proc Natl Acad Sci USA.

[b6] Bloomquist BT, Shortridge RD, Schneuwly S, Perdew M, Montell C, Steller H, Rubin G, Pak WL (1988). Isolation of a putative phospholipase C gene of *Drosophila,* norpA, and its role in phototransduction. Cell.

[b7] Bohm RA, Welch WP, Goodnight LK, Cox LW, Henry LG, Gunter TC, Bao H, Zhang B (2010). A genetic mosaic approach for neural circuit mapping in *Drosophila*. Proc Natl Acad Sci USA.

[b8] Boyden ES, Zhang F, Bamberg E, Nagel G, Deisseroth K (2005). Millisecond-timescale, genetically targeted optical control of neural activity. Nat Neurosci.

[b9] Brand AH, Perrimon N (1993). Targeted gene expression as a means of altering cell fates and generating dominant phenotypes. Development.

[b10] Clyne JD, Miesenbock G (2008). Sex-specific control and tuning of the pattern generator for courtship song in *Drosophila*. Cell.

[b11] Cooley L, Kelley R, Spradling A (1988). Insertional mutagenesis of the *Drosophila* genome with single P elements. Science.

[b12] Dubnau J, Grady L, Kitamoto T, Tully T (2001). Disruption of neurotransmission in *Drosophila* mushroom body blocks retrieval but not acquisition of memory. Nature.

[b13] Fenno L, Yizhar O, Deisseroth K (2011). The development and application of optogenetics. Annu Rev Neurosci.

[b14] Grigliatti TA, Hall L, Rosenbluth R, Suzuki DT (1973). Temperature-sensitive mutations in *Drosophila*
*melanogaster.* XIV. A selection of immobile adults. Mol Gen Genet.

[b15] Hamada FN, Rosenzweig M, Kang K, Pulver SR, Ghezzi A, Jegla TJ, Garrity PA (2008). An internal thermal sensor controlling temperature preference in *Drosophila*. Nature.

[b16] Harcombe ES, Wyman RJ (1977). Outputpattern generation by *Drosophila* flight motoneurons. J Neurophysiol.

[b17] Hotta Y, Benzer S (1969). Abnormal electroretinograms in visual mutants of *Drosophila*. Nature.

[b18] Hotta Y, Benzer S (1970). Genetic dissection of the *Drosophila* nervous system by means of mosaics. Proc Natl Acad Sci US A.

[b19] Hotta Y, Benzer S (1972). Mapping of behaviour in *Drosophila* mosaics. Nature.

[b20] Hotta Y, Benzer S (1976). Courtship in *Drosophila* mosaics: Sex-specific foci for sequential action patterns. Proc Natl Acad Sci US A.

[b21] Ikeda K, Kaplan WD (1970a). Patterned neural activity of a mutant *Drosophilamelanogaster*. Proc Natl Acad Sci US A.

[b22] Ikeda K, Kaplan WD (1970b). Unilaterally patterned neural activity of gynandromorphs, mosaic for a neurological mutant of *Drosophila melanogaster*. Proc Natl Acad Sci US A.

[b23] Ikeda K, Koenig JH, Tsuruhara T (1980). Organization of identified axons innervating the dorsal longitudinal flight muscle of *Drosophila melanogaster*. J Neurocytol.

[b24] Ikeda K, Ozawa S, Hagiwara S (1976). Synaptic transmission reversibly conditioned by single-gene mutation in *Drosophila melanogaster*. Nature.

[b25] Ikeda K, Wiersma CA (1964). Autogenic rhythmicity in the abdominal ganglia of the crayfish: The control of swimmeret movements. Comp Biochem Physiol.

[b26] Ito H, Fujitani K, Usui K, Shimizu-Nishikawa K, Tanaka S, Yamamoto D (1996). Sexual orientation in *Drosophila* is altered by the satori mutation in the sex-determination gene fruitless that encodes a zinc finger protein with a BTB domain. Proc Natl Acad Sci USA.

[b27] Ito K, Okada R, Nobuaki K, Tanaka NK, Awasaki T (2003). Cautionary observations on preparing and interpreting brain images using molecular biology-based staining techniques. Micros Res Tech.

[b28] Kamb A, Iverson LE, Tanouye MA (1987). Molecular characterization of Shaker, a *Drosophila* gene that encodes a potassium channel. Cell.

[b29] Kamikouchi A, Inagaki HK, Effertz T, Hendrich O, Fiala A, Gopfert MC, Ito K (2009). The neural basis *of Drosophila* gravity-sensing and hearing. Nature.

[b30] Kaplan WD, Trout WE (1969). The behavior of four neurological mutants of *Drosophila*. Genetics.

[b31] Kim YT, Wu CF (1990). Allelic interactions at the shibire locus of *Drosophila:* Effects on behavior. J Neurogenet.

[b32] Kimura K, Hachiya T, Koganezawa M, Tazawa T, Yamamoto D (2008). Fruitless and doublesex coordinate to generate male-specific neurons that can initiate courtship. Neuron.

[b33] Kimura K, Ote M, Tazawa T, Yamamoto D (2005). Fruitless specifies sexually dimorphic neural circuitry in the *Drosophila* brain. Nature.

[b34] Kitamoto T (2001). Conditional modification of behavior in *Drosophila* by targeted expression of a temperature-sensitive shibire allele in defined neurons. J Neurobiol.

[b35] Koenig JH, Ikeda K (1980a). Neural interactions controlling timing of flight muscle activity in *Drosophila*. J Exp Biol.

[b36] Koenig JH, Ikeda K (1980b). Interspike interval relationship among flight muscle fibres in *Drosophila*. J Exp Biol.

[b37] Kohatsu S, Koganezawa M, Yamamoto D (2011). Female contact activates male-specific interneurons that trigger stereotypic courtship behavior in *Drosophila*. Neuron.

[b38] Konopka RJ, Benzer S (1971). Clock mutants of *Drosophilamelanogaster*. Proc Natl Acad Sci USA.

[b39] Kosaka T, Ikeda K (1983). Possible temperature-dependent blockage of synaptic vesicle recycling induced by a single gene mutation in *Drosophila*. J Neurobiol.

[b40] Krashes MJ, DasGupta S, Vreede A, White B, Armstrong JD, Waddell S (2009). A neural circuit mechanism integrating motivational state with memory expression in *Drosophila*. Cell.

[b41] Laski RA, Rio DC, Rubin GM (1986). Tissue specificity of *Drosophila* P element transposition is regulated at the level of mRNA splicing. Cell.

[b42] Lee T, Luo L (1999). Mosaic analysis with a repressible cell marker for studies of gene function in neuronal morphogenesis. Neuron.

[b43] Lima SQ, Miesenbock G (2005). Remote control of behavior through genetically targeted photostimulation of neurons. Cell.

[b44] Luan H, Lemon WC, Peabody NC, Pohl JB, Zelensky PK, Wang D, Nitabach MN, Holmes TC, White BH (2006a). Functional dissection of a neuronal network required for cuticle tanning and wing expansion in *Drosophila*. J Neurosci.

[b45] Luan H, Peabody NC, Vinson CR, White BH (2006b). Refined spatial manipulation of neuronal function by combinatorial restriction of transgene expression. Neuron.

[b46] Ma J, Ptashne M (1987). The carboxy-terminal 30 amino acids of GAL4 are recognized by GAL80. Cell.

[b47] Masai I, Hotta Y (1991). Genomic organization of a *Drosophila* phospholipase C, norpA, and molecular lesions in two temperature-sensitive mutants. J Biochem.

[b48] McGuire SE, Le PT, Davis RL (2001). The role of *Drosophila* mushroom body signaling in olfactory memory. Science.

[b49] McGuire SE, Le PT, Osborn AJ, Matsumoto K, Davis RL (2003). Spatiotemporal rescue of memory dysfunction in *Drosophila*. Science.

[b50] Mendel JG (1866). Versuche über Pflanzenhybriden. *Verhandlungen des naturforschenden Vereines in Brünn, Bd*. IV für das Jahr, 1865 Abhandlungen.

[b51] Miesenbock G (2009). The optogenetic catechism. Science.

[b52] Nagel G, Brauner M, Liewald JF, Adeishvili N, Bamberg E, Gottschalk A (2005). Light activation of channelrhodopsin-2 in excitable cells of *Caenorhabditis*
*elegans* triggers rapid behavioral responses. Curr Biol.

[b53] Nagel G, Szellas T, Huhn W, Kateriya S, Adeishvili N, Berthold P, Ollig D, Hegemann P, Bamberg E (2003). Channelrhodopsin-2, a directly light-gated cation-selective membrane channel. Proc Natl Acad Sci USA.

[b54] O'Kane CJ, Gehring WJ (1987). Detection in situ of genomic regulatory elements in *Drosophila*. Proc Natl Acad Sci USA.

[b55] Papazian DM, Schwarz TL, Tempel BL, Jan YN, Jan LY (1987). Cloning of genomic and complementary DNA from Shaker, a putative potassium channel gene from *Drosophila*. Science.

[b56] Peabody NC, Pohl JB, Diao F, Vreede AP, Sandstrom DJ, Wang H, Zelensky PK, White BH (2009). Characterization of the decision network for wing expansion in *Drosophila* using targeted expression of the TRPM8 channel. J Neurosci.

[b57] Pfeiffer BD, Jenett A, Hammonds AS, Ngo TT, Misra S, Murphy C, Scully A, Carlson JW, Wan KH, Laverty TR, Mungall C, Svirskas R, Kadonaga JT, Doe CQ, Eisen MB, Celniker SE, Rubin GM (2008). Tools for neuroanatomy and neurogenetics in *Drosophila*. Proc Natl Acad Sci USA.

[b58] Robertson HM, Preston CR, Phillis RW, Johnson-Schlitz DM, Benz WK, Engels WR (1988). A stable genomic source of P element transposase in *Drosophilamelanogaster*. Genetics.

[b59] Rubin GM, Spradling AC (1982). Genetic transformation of *Drosophila* with transposable element vectors. Science.

[b60] Schroll C, Riemensperger T, Bucher D, Ehmer J, Voller T, Erbguth K, Gerber B, Hendel T, Nagel G, Buchner E, Fiala A (2006). Light-induced activation of distinct modulatory neurons triggers appetitive or aversive learning in *Drosophila* larvae. Curr Biol.

[b61] Spradling AC, Rubin GM (1982). Transposition of cloned P elements into *Drosophila* germ line chromosomes. Science.

[b62] Struhl G, Basler K (1993). Organizing activity of wingless protein in *Drosophila*. Cell.

[b63] Sweeney ST, Broadie K, Keane J, Niemann H, O'Kane CJ (1995). Targeted expression of tetanus toxin light chain in *Drosophila* specifically eliminates synaptic transmission and causes behavioral defects. Neuron.

[b64] Takei K, McPherson PS, Schmid SL, De Camilli P (1995). Tubular membrane invaginations coated by dynamin rings are induced by GTP-gamma S in nerve terminals. Nature.

[b65] Tanouye MA, Wyman RJ (1980). Motor outputs of giant nerve fiber in *Drosophila*. J Neurophysiol.

[b66] Tempel BL, Papazian DM, Schwarz TL, Jan YN, Jan LY (1987). Sequence of a probable potassium channel component encoded at Shaker locus of *Drosophila*. Science.

[b67] Turrigiano GG, Nelson SB (2000). Hebb and homeostasis in neuronal plasticity. Curr Opin Neurobiol.

[b68] van der Bliek AM, Meyerowitz EM (1991). Dynamin-like protein encoded by the *Drosophila* shibire gene associated with vesicular traffic. Nature.

[b69] von Philipsborn AC, Liu T, Yu JY, Masser C, Bidaye SS, Dickson BJ (2011). Neuronal control of *Drosophila* courtship song. Neuron.

[b70] Waddell S, Armstrong JD, Kitamoto T, Kaiser K, Quinn WG (2000). The amnesiac gene product is expressed in two neurons in the *Drosophila* brain that are critical for memory. Cell.

[b71] White BH, Osterwalder TP, Yoon KS, Joiner WJ, Whim MD, Kaczmarek LK, Keshishian H (2001). Targeted attenuation of electrical activity in *Drosophila* using a genetically modified K(+) channel. Neuron.

[b72] Wiersma CA, Ikeda K (1964). Interneurons Commanding Swimmeret Movements in the Crayfish, *Procambarus clarki* (Girard). Comp Biochem Physiol.

[b73] Yoshihara M, Adolfsen B, Galle KT, Littleton JT (2005). Retrograde signaling by Syt 4 induces presynaptic release and synapse-specific growth. Science.

[b74] Yoshihara, Ito (2000). Improved Gal4 screening kit for large-scale generation of enhancer-trap strains. Dros InfSer.

[b75] Yoshihara M, Takasu-Ishikawa E, Sakai Y, Hotta Y, Okamoto H (1988). Single P-element mutagenesis in *Drosophila melanogaster*. Proc Jpn Acad.

[b76] Yoshihara M, Ueda A, Zhang D, Deitcher DL, Schwarz TL, Kidokoro Y (1999). Selective effects of neuronal-synaptobrevin mutations on transmitter release evoked by sustained versus transient Ca^2+^ increases and by cAMP. J Neurosci.

[b77] Zhang F, Wang LP, Boyden ES, Deisseroth K (2006). Channelrhodopsin-2 and optical control of excitable cells. Nat Methods.

